# Ubiquitin-Specific-Processing Protease 7 Regulates Female Germline Stem Cell Self-Renewal Through DNA Methylation

**DOI:** 10.1007/s12015-020-10076-9

**Published:** 2020-11-05

**Authors:** Yongqiang Zhao, Xiaoyong Li, Geng Tian, Xinyan Zhao, Jiemin Wong, Yue Shen, Ji Wu

**Affiliations:** 1 Renji Hospital, Key Laboratory for the Genetics of Developmental and Neuropsychiatric Disorders (Ministry of Education), Bio-X Institutes, School of Medicine, Shanghai Jiao Tong University, Shanghai, 200240 China; 2 Key Laboratory of Fertility Preservation and Maintenance of Ministry of Education, Ningxia Medical University, Yinchuan, 750004 China; 3 Shanghai Key Laboratory of Regulatory Biology, Fengxian District Central Hospital-ECNU Joint Center of Translational Medicine, Institute of Biomedical Sciences and School of Life Sciences, East China Normal University, Shanghai, 200241 China

**Keywords:** *Usp7*, FGSCs, DNA methylation, Histone modification, Self-renewal, Differentiation

## Abstract

Ubiquitin-specific-processing protease 7 (*Usp7*) is a key deubiquitinase controlling epigenetic modification and regulating the self-renewal, proliferation, and differentiation of stem cells. However, the functions and mechanisms of action of *Usp7* on female germline stem cells (FGSCs) are unknown. Here, we demonstrated that *Usp7* regulated FGSC self-renewal via DNA methylation. The results of Cell Counting Kit-8 and 5-ethynyl-20-deoxyuridine assays showed that the viability and proliferation of FGSCs were negatively regulated by *Usp7*. Moreover, *Usp7* downregulated the expression of self-renewal genes, such as *Oct4*, *Etv5*, *Foxo1*, and *Akt*, but upregulated the expression of differentiation-related genes including *Stra8* and *Sycp3.* Mechanistically, RNA-seq results showed that *Usp7* negatively regulated FGSC self-renewal but positively modulated differentiation in FGSCs. Meanwhile, both overexpression and knockdown of *Usp7* resulted in significant changes in DNA methylation and histone modification in FGSCs. Additionally, RNA-seq and MeDIP-seq analyses showed that *Usp7* regulates the self-renewal and differentiation of FGSCs mainly through DNA methylation rather than histone modification, which was also confirmed by a rescue assay. Our study not only offers a novel method to research FGSC self-renewal and differentiation in view of epigenetic modifications, but also provides a deep understanding of FGSC development.

**Supplementary Information:**

The online version contains supplementary material available at 10.1007/s12015-020-10076-9.

**Figure:**
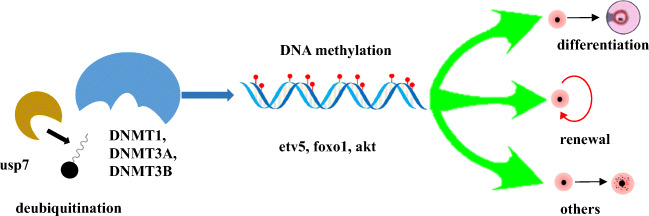
Graphical Abstract

## Introduction

It has been reported that about 48.5 million couples suffer from infertility worldwide [28]. Disorders in oogenesis are the main cause of female infertility. Oogenesis is a highly complex process that is intricately regulated by the interactions of multiple genes and various signaling molecules, which makes the investigation of oogenesis disorders extremely challenging. Many risk factors, such as genetic, epigenetic, and environmental ones, result in oogenesis disorders. In recent years, the discovery of female germline stem cells (FGSCs) has provided a new perspective for the treatment of such diseases [41, 42, 50]. As the germline stem cells that supplement oocytes, FGSCs can maintain the reproductive function of the ovary, indirectly maintain the endocrinological status of the ovary, and delay premature ovarian failure. They are of great significance for improving the quality of follicles and the pregnancy rate of female mammals and are becoming a particular focus of the medical community.

Several studies have revealed the mechanism underlying FGSC self-renewal, differentiation, and autophagy [19, 46, 51, 52]. Additionally, FGSCs were demonstrated to have the potential to differentiate into oocytes, which provided a new strategy to research oogenesis [7, 41, 52]. Comparing FGSCs and spermatogonial stem cells (SSCs), Li et al. uncovered the transcript structures, genetic variants, and interaction between microRNAs and circRNAs [20]. Li et al. suggested that chemical compounds play important roles in modulating FGSCs via the PI3K/Akt pathway [19]. Additionally, Ma et al. revealed that *Etv5*, *Foxo1*, and *Akt* genes positively regulate FGSC self-renewal [27]. Epigenetic research on the development of stem cells, especially germ stem cells, has become a hotspot and a field in which many outstanding results were achieved [12, 18]. Zhang et al. revealed that DNA methylation contributes to the unipotency of FGSCs and is involved in the maintenance of FGSC sexual identity [49]. These studies established the theoretical foundations for researching the regulatory mechanism of FGSCs and provided a deep understanding of germ cell development.

Ubiquitin-specific-processing protease 7 (*Usp7*) is a well-known deubiquitinase. It is widely expressed and works by stabilizing substrate proteins [31, 34]. *Usp7* removes ubiquitin from multiple protein substrates and participates in a variety of cellular processes, including immune response, mitosis, and DNA repair [1, 2, 24, 30, 34]. Recent studies have shown that *Usp7* is involved in cell differentiation. For example, it is required for the osteogenic differentiation of human adipose-derived stem cells [36]. Additionally, Liang et al. revealed that *Usp7* regulates human terminal erythroid differentiation [22] and promotes the invasion and metastasis of cancer [25]. Deubiquitination is the basic function of *Usp7* and its epigenetic regulation has become a new research hotspot. USP7 participates in the regulation of methylation through interacting with DNMT1, DNMT3a, DNMT3b, and UHRF1 [4, 9]. Additionally, *Usp7* had a major effect on the progress of histone modification [23, 40]. Histone modification is an important way in which *Usp7* regulates cellular functions [10, 37, 38]. *Usp7* is now a key molecule in epigenetic research and has been a focus of ongoing studies. However, to the best of our knowledge, no studies have evaluated the mechanism and function of *Usp7* in determining FGSC self-renewal and differentiation.

In this paper, we report for the first time that *Usp7* inhibited the proliferation of FGSCs and promoted their differentiation via genomic methylation. This study provides a better understanding of the regulatory mechanism of FGSC self-renewal and differentiation, and might provide a theoretical basis for improving the diagnosis and treatment of ovarian dysfunction and other related diseases.

## Results

### Characterization of Female Germline Stem Cells

**Fig. 1:**
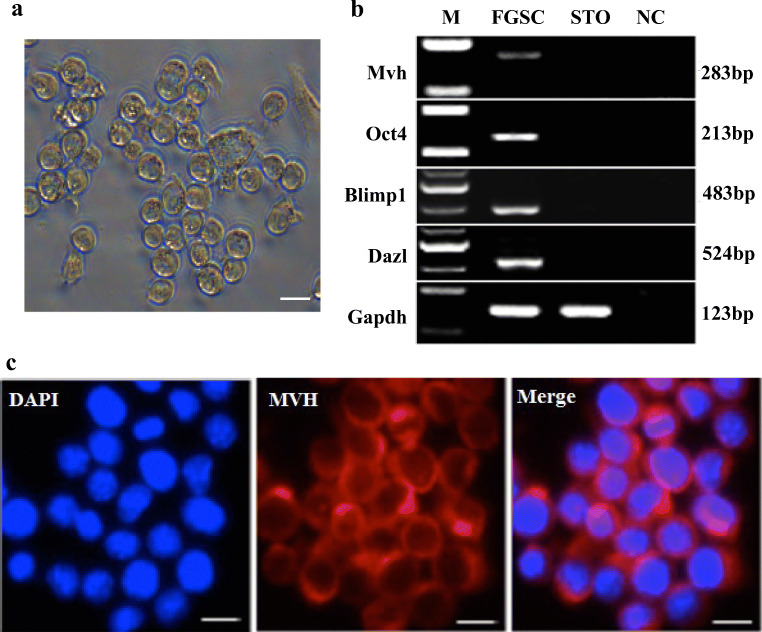
Characteristics of female germline stem cells. (**a**) Representative microscopy images of the morphological characteristics of female germline stem cells (FGSCs). (**b**) Reverse-transcription PCR identification of *Mvh*, *Oct4*, *Blimp1*, and *Dazl* mRNA expression in FGSCs. M, 250 bp DNA markers. Gapdh serves as an endogenous control for normalization. *NC* negative control. (**c**) Immunofluorescence staining of MVH in FGSCs (*n* = 3 per group). Scale bars: 10 μm in (**a**) and (**c**)

To characterize the FGSCs used in this study (Fig. 1a), we identified marker genes of germ cells: *Oct4* (also known as *Pou5f1*, POU domain, class 5, transcription factor 1) [32], *Mvh* [also known as *Ddx4*, DEAD (Asp-Glu-Ala-Asp) box polypeptide 4] [6], *Blimp1* (also known as *prdm1*, PR domain containing 1) [29], and *Dazl* (deleted in azoospermia-like) [45]. The expression of *Oct4*, *Mvh*, *Blimp1*, and *Dazl* was confirmed by RT-PCR (Fig. 1b). The positivity of MVH was further identified by immunofluorescence analysis (Fig. 1c). These obtained results suggested that the FGSCs used were standardized.

### Usp7 Is Upregulated during the Differentiation of Female Germline Stem Cells Induced by Retinoic Acid and Granulosa Cells

**Fig. 2:**
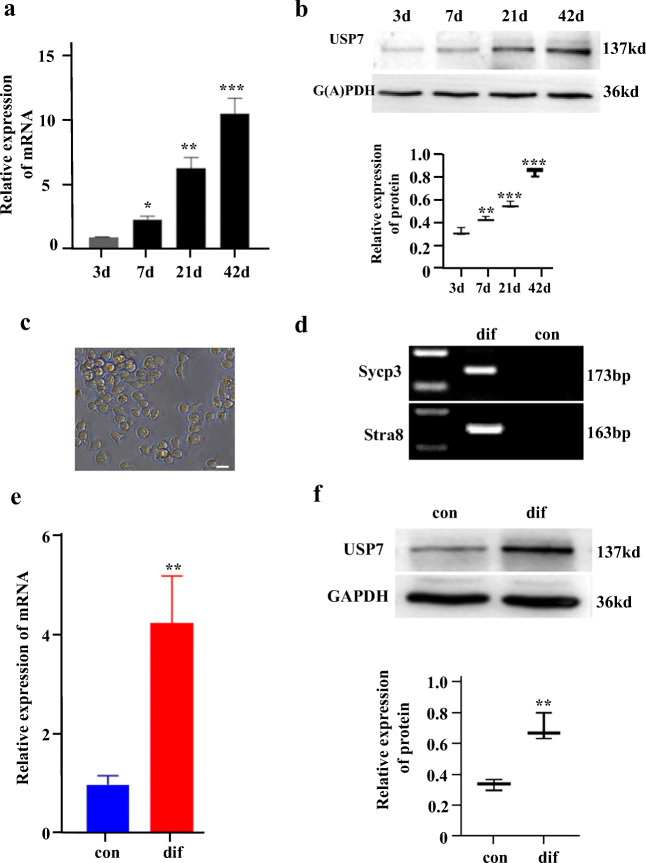
*Usp7* expression is upregulated in differentiated female germline stem cells. (**a**, **b**) Relative expression of *Usp7* mRNA (**a**) and protein (**b**) in the ovary of postnatal mice at various times. (**c**) Microscopic observation of the morphological characteristics of in vitro differentiated FGSCs. (**d**) RT-PCR identification of *Sycp3* and *Stra8* in FGSCs differentiated in vitro. (**e**, **f**) Relative expression of *Usp7* mRNA (**e**) and protein (**f**) in FGSCs differentiated in vitro. Values are mean ± SD (n = 3). *P < 0.05, **P < 0.01, ***P < 0.001. *3d* 3 days, *7d* 7 days, *21d* 21 days, *42d* 42 days. *Dif* in vitro differentiated FGSCs, con: FGSCs

To study the involvement of *Usp7* in the development of FGSCs, we first examined its expression in the ovary of postnatal mice at various times (3, 7, 21, and 42 days) by qRT-PCR and western blotting (Fig. 2a, b). The results showed that the expression of *Usp7* gradually increased in the process of ovarian development. This indicated that *Usp7* plays an important role in FGSC development. qRT-PCR and western blotting were then performed to analyze the expression of *Usp7* during the in vitro differentiation of FGSCs induced by retinoic acid and granulosa cells [7, 52]. The results showed that the expression of *Usp7* was increased in differentiated FGSCs (Fig. 2c–2f). We deduced that *Usp7* is upregulated during the differentiation of FGSCs.

### Knockdown of Usp7 Enhances Female Germline Stem Cell Growth and Self-Renewal

**Fig. 3:**
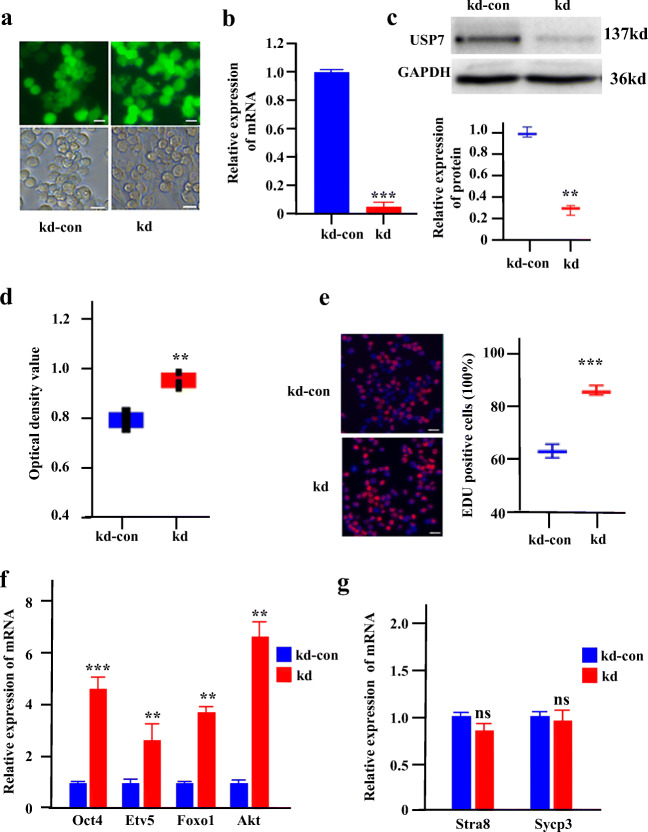
Knockdown of *Usp7* enhances female germline stem cell growth and self-renewal. (**a**) Fluorescence and bright field image of FGSCs infected with lentivirus. (**b**) qRT-PCR examined the expression level of *Usp7* in FGSCs infected with *Usp7*-knockdown lentivirus or *Usp7-*knockdown lentivirus control. (**c**) Top: Western blot analyses of the levels of USP7 in FGSCs infected with lentivirus. Bottom: Statistical results of western blotting. (**d**) CCK-8 assays were performed using FGSCs infected with *Usp7-*knockdown lentivirus. (**e**) EdU assays were performed using FGSCs infected with *Usp7*-knockdown lentivirus. (**f**) The expression of *Oct4*, *Etv5*, *Foxo1*, and *Akt* in *Usp7*-knockdown FGSCs. (**g**) The expression of *Strat8* and *Sycp3* in *Usp7-*knockdown FGSCs. Scale bars: 10 μm in (**a**); 20 μm in (**e**). *P < 0.05;**P < 0.01; ***P < 0.001. kd-con: *Usp7* knockdown control, kd: *Usp7* knockdown

To further investigate the function of *Usp7* in FGSCs, we knocked down *Usp7* using shRNAs (Fig. 3a). The results of *Usp7* knockdown in FGSCs were confirmed by qRT-PCR and western blotting. Knockdown of *Usp7* resulted in more than 75% and 50% decreases in mRNA (Fig. 3b) and protein levels (Fig. 3c), respectively, compared with those in the control. We next examined the effect of *Usp7* knockdown on the proliferation of FGSCs using CCK8 assay (Fig. 3d) and EdU incorporation assay (Fig. 3e). The results showed that knockdown of *Usp7* in FGSCs significantly improved their proliferation compared with that of controls. Accordingly, the expression of *Oct4*, *Etv5*, *Foxo1*, and *Akt*, genes related to FGSC self-renewal, was upregulated in *Usp7*-knockdown FGSCs (Fig. 3f). Meanwhile, *Stra8* and *Sycp3*, differentiation-related genes, showed no significant changes in *Usp7-*knockdown FGSCs compared with the levels in the corresponding control (Fig. 3g). These results together indicate that the knockdown of *Usp7* enhanced FGSC growth and self-renewal.

### Usp7 Overexpression Inhibits Female Germline Stem Cell Growth and Self-Renewal, while Improving Female Germline Stem Cell Differentiation

**Fig. 4:**
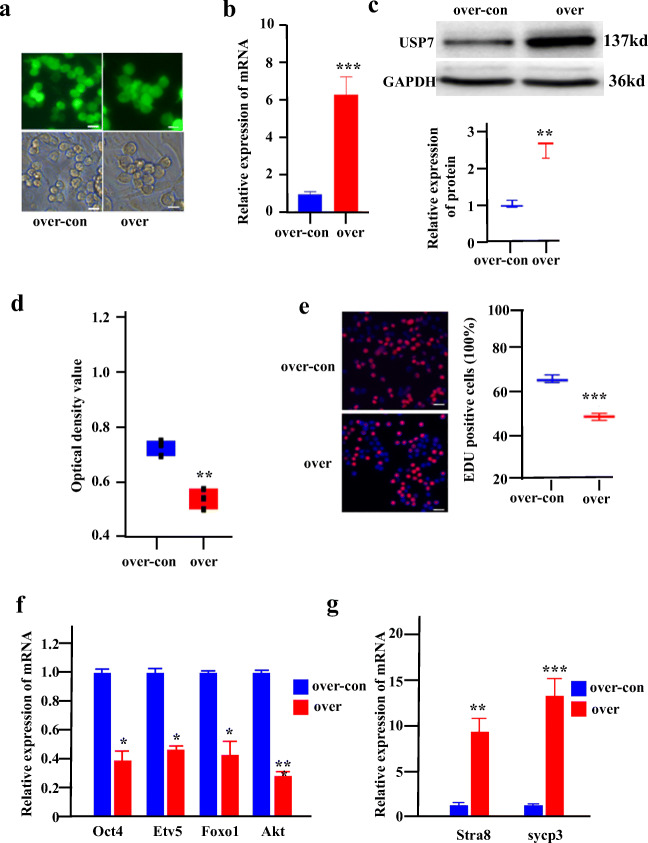
*Usp7* overexpression inhibits female germline stem cell growth and self-renewal, while improving FGSC differentiation. (**a**) Fluorescence and bright field image for FGSCs infected with lentivirus. (**b**) qRT-PCR examined the expression level of *Usp7* in FGSCs infected with *Usp7-*overexpressing lentivirus or *Usp7-*overexpressing lentivirus control. (**c**) Top: Western blot analyses of the levels of USP7 in FGSCs infected with lentivirus. Bottom: Statistical results of western blotting. (**d**) CCK-8 assays were performed using FGSCs infected with *Usp7-*overexpressing lentivirus. (**e**) EdU assays were performed using FGSCs infected with *Usp7-*overexpressing lentivirus. (**f**) The expression of *Oct4*, *Etv5*, *Foxo1*, and *Akt* in *Usp7-*overexpressing FGSCs. (**g**) The expression of *strat8* and sycp3 in *Usp7*-overexpressing FGSCs. Scale bars: 10 μm in (**a**); 20 μm in (**e**). *P < 0.05;**P < 0.01; ***P < 0.001. over-con: *Usp7* overexpression control, over: *Usp7* overexpression

We next investigated how upregulated *Usp7* affects the growth and self-renewal of FGSCs by overexpressing *Usp7*. The FGSC line was transfected with lentivirus expressing control vector and *Usp7*. The overexpression of *Usp7* was confirmed by qRT-PCR and western blotting. This overexpression led to >6-fold and > 2.5-fold increases in mRNA (Fig. 4b) and protein levels (Fig. 4c) in FGSCs compared with those in control cells. The results showed that the overexpression of *Usp7* in FGSCs significantly inhibited their proliferation compared with that of controls (Fig. 4d, e). Accordingly, the expression of *Oct4*, *Etv5*, *Foxo1*, and *Akt* was downregulated in *Usp7-*overexpressing FGSCs (Fig. 4f). Meanwhile, we found that marker genes related to differentiation, namely, *Stra8* and *Sycp3*, were upregulated in *Usp7-*overexpressing FGSCs compared with those in their control (Fig. 4g). Taking these findings together, the overexpression of *Usp7* inhibits FGSC growth and self-renewal and improves FGSC differentiation.

### Usp7 Regulates Female Germline Stem Cell Self-Renewal and Differentiation as Revealed by RNA-Seq

**Fig. 5:**
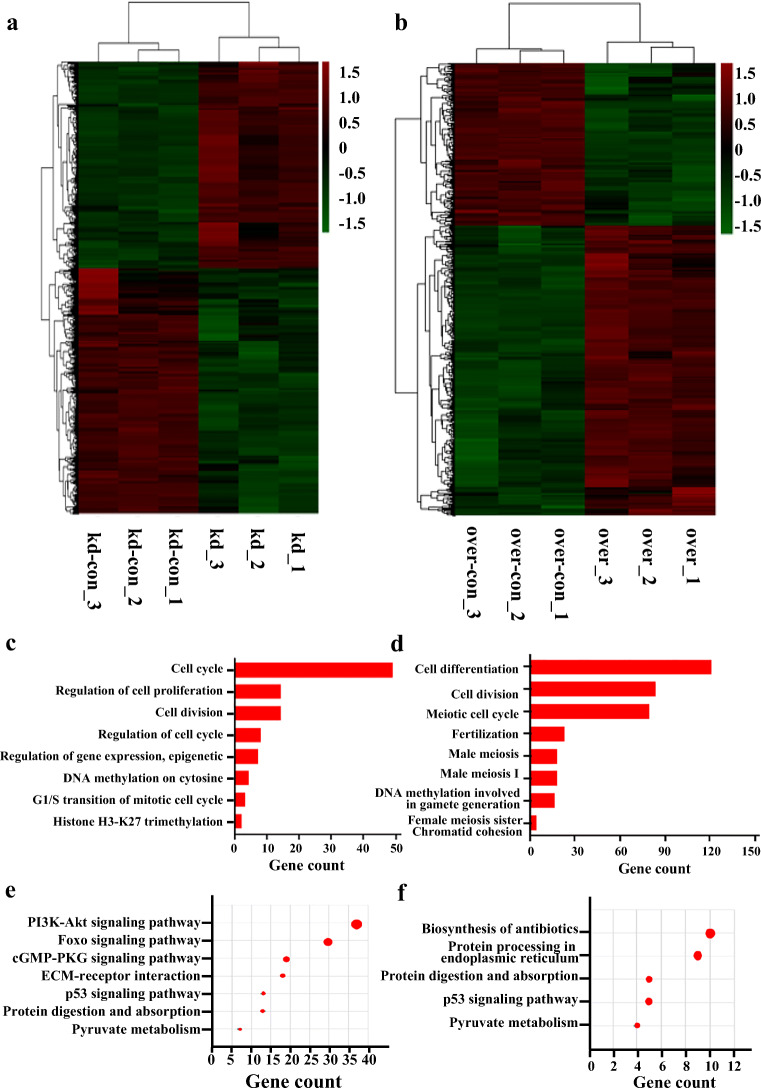
RNA-seq reveals that *Usp7* alters female germline stem cell renewal and differentiation. (**a**, **b**) Hierarchical clustering shows differentially expressed mRNA patterns between *Usp7-*knockdown FGSCs (**a**) and *Usp7-*overexpressing FGSCs (**b**) compared with the corresponding controls. (**c**, **d**) GO analysis of differentially expressed genes in *Usp7-*knockdown (**c**) and -overexpressing FGSCs (**d**) compared with the corresponding controls. GO analysis was performed by DAVID. Differentially expressed genes had an adjusted *P* < 0.01 and a twofold or greater difference in expression. (**e**, **f**) KEGG pathway terms of differentially expressed genes in *Usp7-*knockdown (**e**) and -overexpressing FGSCs (**f**). KEGG pathway analysis was performed by DAVID

To elucidate the mechanisms underlying *Usp7*’s regulation of the self-renewal and differentiation of FGSCs, we performed RNA-seq of FGSCs with *Usp7* knockdown and overexpression. Fast-QC was carried out to ensure the data quality (Fig. S1a–S1d). For each replicate, a Q-score higher than 30 (error < 0.1%) was considered to suggest that the sequencing results were reliable. Overall, a total of 1044.2 million reads were generated. After applying a stringent filtering approach, 3802 differentially expressed genes were detected (adjusted *p* value <0.05; fold change >2). There were 414 upregulated and 232 downregulated genes in *Usp7*-overexpressing FGSCs compared with the levels in their control, while there were 1446 upregulated and 1710 downregulated genes in the *Usp7-*knockdown FGSCs compared with the levels in their control. The differentially expressed mRNAs were calculated by MeV_4_9_0 cluster analysis to generate heat maps (Fig. 5a, b) and volcano plots (Fig. S2a, S2b). Some genes were also selected at random to verify the results of RNA-seq by qRT-PCR (Fig. S2c).

The differentially expressed genes were further analyzed with the Database for Annotation, Visualization and Integrated Discovery (DAVID; http://david.abcc.ncifcrf.gov) [13, 27]. Gene Ontology (GO) and KEGG pathway analyses were used to determine the biological process terms particularly associated with the differentially expressed genes. The GO and KEGG pathway terms are shown in Fig. 5c–5f. GO terms in *Usp7-*knockdown FGSCs and their control showed particular associations with the cell cycle, regulation of cell proliferation, and cell division. Meanwhile, cell differentiation and fertilization were particularly associated with the *Usp7-*overexpressing FGSCs and their control. Moreover, DNA methylation and histone H3-K27 trimethylation were particularly identified, which reveals that *Usp7* may modulate FGSCs via DNA methylation or histone modification.

Furthermore, KEGG analysis terms in the *Usp7-*knockdown FGSCs and their control showed particular associations with the PI3K-Akt signaling pathway, Foxo signaling pathway, and cGMP-PKG signaling pathway. Meanwhile KEGG analysis terms in the *Usp7-*overexpressing FGSCs and their control showed particular associations with protein digestion and absorption, p53 signaling pathway, and other pathways. These biological processes and pathways were consistent with our CCK8, EdU, and qRT-PCR results. In particular, the PI3K-Akt signaling pathway was confirmed to regulate FGSCs [19, 27], which meant that *Usp7* may modulate FGSCs via the PI3K-Akt signaling pathway.

### Usp7 Affects Female Germline Stem Cell Development through DNA Methylation

**Fig. 6:**
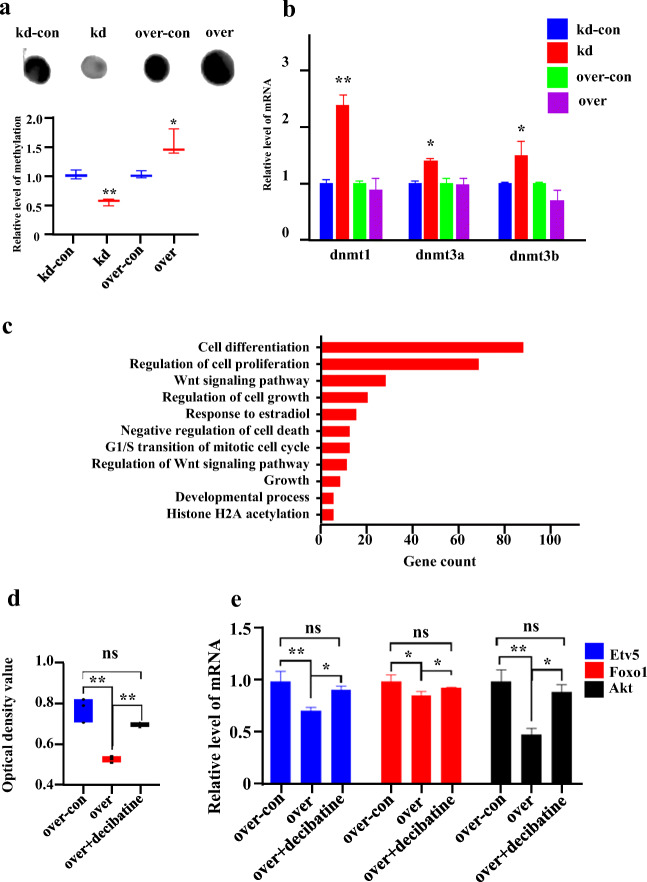
*Usp7* affects female germline stem cell development through DNA methylation to modulate FGSCs. (**a**) Dot blotting was performed to identify the DNA methylation level in *Usp7-*knockdown control, −knockdown, −overexpressing control, and -overexpressing FGSCs. (**b**) Relative expression of dnmt1, dnmt3a, and dnmt3b at the mRNA level in *Usp7*-knockdown control, −knockdown, −overexpressing control, and -overexpressing FGSCs. (**c**) GO analysis of the genes showing different DNA methylation patterns in *Usp7-*knockdown FGSCs and their control. (**d**) CCK8 showing increased optical density value in *Usp7-*overexpressing FGSCs treated with the DNA methylation inhibitor decitabine. (**e**) qRT-PCR showing the expression of *Etv5*, *Foxo1*, and *Akt* genes in *Usp7-*overexpressing FGSCs treated with the DNA methylation inhibitor decitabine. *P < 0.05, **P < 0.01, ***P < 0.001. *ns* no significant difference

To determine whether *Usp7* modulates FGSCs via DNA methylation, dot blotting was performed (Fig. 6a). The results showed that DNA methylation is positively correlated with *Usp7* in FGSCs. The mRNA expression of *Dnmt1*, *Dnmt3a*, and *Dnmt3b*, known as methyltransferase genes [47], was analyzed using qRT-PCR (Fig. 6b). However, the mRNA expression of these DNA methyltransferases was increased in *Usp7-*knockdown FGSCs but showed no significant changes in *Usp7-*overexpressing FGSCs. We deduced that *Usp7* deubiquitinated these target proteins and protected them from degradation, which thus led to compensatory increases in the mRNA levels.

Furthermore, we determined the genomic DNA methylation with methylated DNA immunoprecipitation sequence (MeDIP-seq) in *Usp7-*knockdown FGSCs and their control. Fast-QC was carried out to ensure the data quality (Fig. S3a–S3b). A total of 315.2 million reads yielding 38.1 million peaks were generated. Overall, 2059 genes were screened and showed different DNA methylation patterns. These 2059 genes were subjected to GO and KEGG pathway analyses. The GO terms are shown in Fig. 6c and KEGG terms in Fig. S4a. The GO terms showed particular associations with cell differentiation, regulation of cell proliferation, regulation of cell growth, and negative regulation of cell growth. The KEGG pathway terms showed particular associations with the PI3K-Akt signaling pathway, MAPK signaling pathway, and Wnt signaling pathway. These GO and KEGG terms from the DNA methylation data reflected those from the RNA-seq analysis.

Additionally, upon comparing these 2059 genes in genomic DNA methylation and differentially expressed genes in RNA-seq, we identified 255 overlapping genes. GO analysis was carried out again to further reveal the functions of DNA methylation. This showed that cell differentiation, regulation of cell proliferation, and regulation of gene expression were particularly associated with the GO terms (Fig. S4b). These results suggested that changes of DNA methylation may result in changes in gene expression, followed by the modulation of FGSCs. Thus, we speculated that *Usp7* may regulate the FGSCs by genomic methylation.

To determine how *Usp7* regulates the FGSCs by genomic methylation, decitabine, a DNA methyltransferase inhibitor, was used to identify whether inhibitor treatment of *Usp7-*overexpressing FGSCs would rescue cell defects. Dot blotting confirmed that the level of genomic methylation was improved in *Usp7-*overexpressing FGSCs. Therefore, the *Usp7-*overexpressing FGSCs were treated with decitabine for 48 h. Decitabine treatment transiently decreased the expression level of *Dnmt1*, *Dnmt3a*, and *Dnmt3b* as detected by RT-qPCR, indicating that the inhibitors worked as expected (Fig. S5). After treatment, the CCK8 assay results showed that the viability value was increased from 0.524 to 0.695, which was decreased from 0.776 to 0.524 by *Usp7* overexpression in FGSCs (Fig. 6d). Meanwhile, qRT-PCR results showed that the gene expression of *Etv5*, *Foxo1*, and *Akt* was improved at the mRNA level after treatment with decitabine (Fig. 6e). These observations suggested that inhibiting the process of DNA methylation rescued the growth defects of *Usp7-*overexpressing FGSCs. Thus, we deduced that *Usp7* regulates FGSCs via the regulation of genomic DNA methylation. These results suggest that *Usp7* regulates FGSC proliferation and differentiation through DNA methylation.

### Usp7 Affects Female Germline Stem Cell Development, but Not through Histone Modification

**Fig. 7:**
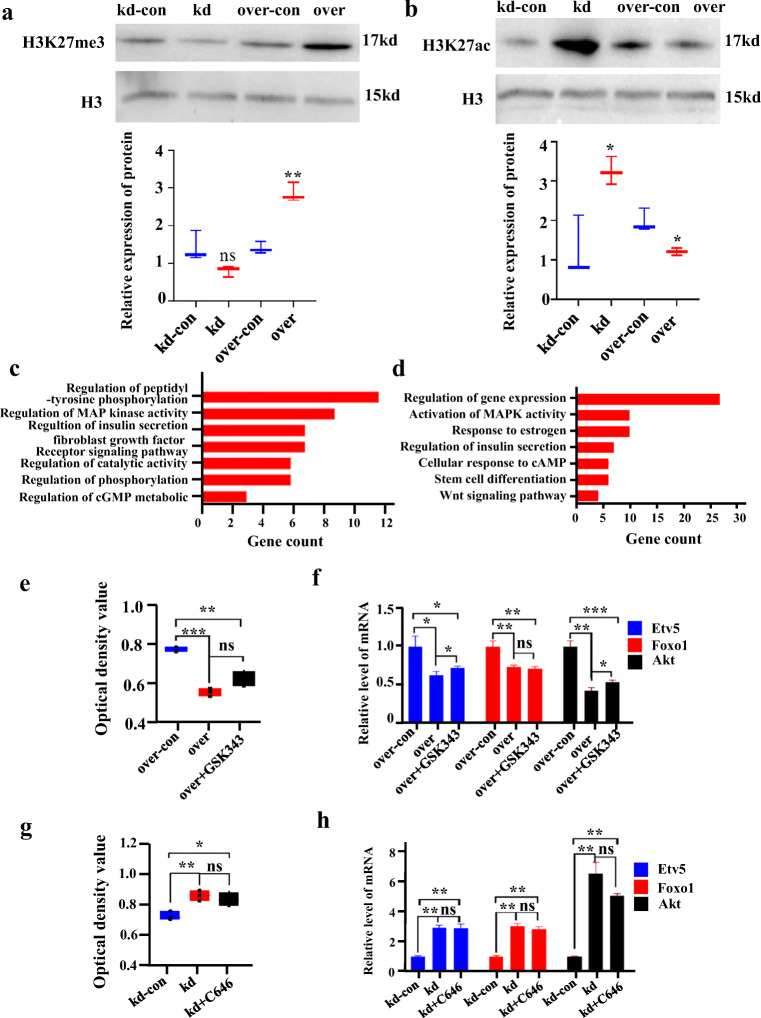
*Usp7* does not regulate female germline stem cell development through histone modification. (**a**, **b**) Representative western blots of acid-extracted histones from FGSCs using antibodies recognizing H3K27me3 (**a**) and H3K27ac (**b**). The band sizes range from 17 to 23 kDa, as expected for modified histones. (**c**, **d**) GO analysis of the genes showing differential H3K27me3 (**c**) and H3K27ac (**d**) modification patterns in *Usp7-*knockdown FGSCs and their control. (**e**) Cell growth analysis based on a CCK8 assay of FGSCs treated with the H3K27me3 inhibitor GSK343. The absorbance ratio of the overexpression group relative to controls is shown. (**f**) qRT-PCR showing the increased expression of *Etv5*, *Foxo1*, and *Akt* genes in *Usp7-*overexpressing versus control FGSCs treated with the H3K27me3 inhibitor GSK343. (**g**) Cell growth analysis based on a CCK8 assay of FGSCs treated with the H3K27ac inhibitor C646. The absorbance ratio of the *Usp7-*knockdown group relative to controls is shown. (**h**) qRT-PCR showing no obvious increase in the expression of *Etv5*, *Foxo1*, and *Akt* genes in *Usp7-*knockdown versus control FGSCs treated with the H3K27ac inhibitor C646. *P < 0.05, **P < 0.01, ***P < 0.001. *ns* no significant difference

To determine whether *Usp7* modulates FGSCs through histone modification, western blotting was used to determine the changes of H3K27me3 and H3K27ac, and confirmed that *Usp7* positively regulates the level of H3K27me3 (Fig. 7a), but negatively controls H3K27ac (Fig. 7b). We also tested the effect of *Usp7* on H3K9me3, and the results showed that *Usp7* did not regulated the level of H3K9me3 (Fig. S6). We then performed H3K27me3 and H3K27ac ChIP-seq analysis in *Usp7-*knockdown FGSCs and their control. Fast-QC was carried out to ensure the data quality (Fig. S7a–S7f). A total of 1065.1 million reads were generated. Overall, 1563 and 1151 genes showed different H3K27me3 and H3K27ac modification patterns in *Usp7-*knockdown FGSCs compared with their control. These 1563 genes from H3K27me3 ChIP-seq were subjected to GO and KEGG pathway analyses. The GO terms are shown in Fig. 7c and KEGG terms in Fig. S6a. The GO analysis suggested particular associations with the regulation of MAP kinase activity, fibroblast growth factor receptor signaling pathway, and regulation of cGMP metabolic process. KEGG analysis suggested particular associations with the Wnt signaling pathway, thyroid hormone signaling pathway, and chemical carcinogenesis. These 1151 genes from H3K27ac ChIP-seq were subjected to GO and KEGG pathway analyses. The GO terms are shown in Fig. 7d and KEGG terms in Fig. S6b. The GO analysis suggested particular associations with the regulation of gene expression, activation of MAPK activity, and Wnt signaling pathway. The KEGG analysis suggested particular associations with the MAPK signaling pathway, Ras signaling pathway, and cAMP signaling pathway.

Additionally, upon comparing these 1563 genes in the H3K27me3 ChIP-seq data and the differentially expressed genes in RNA-seq, we identified 213 overlapping genes. The GO analysis terms showed particular associations with the regulation of peptidyl-tyrosine phosphorylation, regulation of MAP kinase activity, and regulation of catalytic activity (Fig. S8c). Meanwhile, upon comparing these 1151 genes in the H3K27ac ChIP-seq data and the differentially expressed genes in RNA-seq, we identified 155 overlapping genes. The GO analysis results are shown in Fig. S6d. The GO analysis terms showed particular associations with nucleosome assembly, cell-cell adhesion, and regulation of cell death.

To identify the actual roles of H3K27me3 and H3K27ac in FGSCs, histone methyltransferase inhibitor (GSK343), which represses EZH2-dependent H3K27me3 formation, and histone acetyltransferase inhibitor (C646), which restrains the activity of the H3K27 acetyltransferase crebbp (CBP)/p300, were used to treat the *Usp7-*overexpressing FGSCs and *Usp7-*knockdown FGSCs to carry out a rescue assay. GSK343 treatment transiently decreased the expression level of Ezh2, and GSK343 treatment transiently decreased the expression level of (CBP)/p300, indicating that the inhibitors worked as expected (Fig. S9). After the FGSCs had been treated with GSK343, CCK8 and qRT-PCR were performed again. The results showed that although *Etv5* and *Akt* were increased by GSK343 to some extent, the rescue effect was insufficient (Fig. 7e, f). Meanwhile, the results of CCK8 and qRT-PCR showed no changes after treatment with C646 (Fig. 7g, h). We deduced that *Usp7* regulates FGSCs at least in part through histone methylation, but this is not the key mechanism determining FGSC fate.

## Discussion

Based on the above datas, we propose a model in which *Usp7* alters DNA methylation by interacting with target proteins, such as DNMT1, DNMT3A, and DNMT3B, and modulates the DNA methylation level of genes related to the self-renewal and differentiation of FGSCs. This in turn results in a change in the fate determination of FGSCs.

In this study, we observed that the expression of *Usp7* was increased in ovary development by using qRT-PCR and western blotting on the ovaries of mice of different ages. The increased expression suggested that *Usp7* may be involved in differentiation. It was identified by detecting differentiated FGSCs. The results are consistent with previous findings [36]. Meanwhile, accumulated research has shown that *Usp7* plays a vital role in cell development. Our results showed that *Usp7* overexpression negatively regulates FGSC self-renewal but positively modulates differentiation in FGSCs. This corresponds to the findings from the experiment in ovaries [Fig. 2a, b] and human adipose-derived stem cells [36]. Next, we performed RNA sequencing to elucidate the underlying mechanisms by which *Usp7* regulates FGSC development. GO and KEGG analyses showed particular associations with the regulation of cell proliferation and cell division in *Usp7-*knockdown FGSCs. Meanwhile, cell differentiation was particularly identified in the *Usp7*-overexpressing FGSCs. RNA-seq confirmed the findings of our previous assay. Additionally, given that DNA methylation was particularly identified among the GO terms and *Usp7* regulated the genomic methylation and modulated cell fate, a dot blot on FGSC DNA methylation was performed. This revealed that *Usp7* positively regulates DNA methylation in FGSCs. However, interestingly but confusingly, the mRNA expression of methyltransferases such as *Dnmt1*, *Dnmt3a*, and *Dnmt3b* was upregulated in *Usp7-*knockdown FGSCs. However, there were hardly any changes in the *Usp7-*overexpressing FGSCs. We deduced that *Usp7* knockdown resulted in the increased degradation of these methyltransferases, which led to compensatory increases in mRNA levels. Based on the dot blot assay, MeDIP-seq was performed. The GO terms showed particular associations with cell differentiation and proliferation, which was consistent with the RNA-seq data and cell assay results. We deduced that *Usp7* regulates FGSCs through DNA methylation. To confirm this, decitabine, a DNA methyltransferase inhibitor, was used to carry out a rescue assay. The results of CCK8 and qRT-PCR suggested that decitabine rescued cell defects. This means that *Usp7* regulates FGSCs via DNA methylation. Moreover, H3K27 trimethylation was particularly identified in the RNA-seq GO analysis (Fig. 4d) and substantial research has confirmed that *Usp7* regulates cell fate through histone modification [10, 14, 17]. H3K27me3 is associated with gene silencing and H3K27ac is associated with gene activation [43, 44]. Thus, we performed western blotting to determine the relationship between H3K27me3 or H3K27ac levels and *Usp7* expression in FGSCs. The western blotting results suggested that *Usp7* positively regulates the level of H3K27me3, but negatively controls that of H3K27ac. Therefore, we performed H3K27me3 and H3K27ac ChIP-seq analysis in *Usp7-*knockdown FGSCs and their control. The GO terms showed particular associations with the regulation of MAP kinase activity in the H3K27me3 ChIP-seq data. Meanwhile, gene expression, cell proliferation, activation of MAPK activity, and stem cell differentiation were particularly identified in the H3K27ac ChIP-seq data. Similarly, histone methyltransferase inhibitor (GSK343) and histone acetyltransferase inhibitor (C646) were each used to carry out rescue assays. qRT-PCR suggested that *Etv5* and *Akt* were partly upregulated by GSK343. However, the rescue effect was insufficient. Meanwhile, there were no obvious changes after treatment with C646. Additionally, upon comparing the GO terms in H3K27me3 or H3K27ac ChIP-seq and RNA-seq, there were still enormous changes. Thus, we deduced that *Usp7* regulates FGSCs though DNA methylation. The PI3K/Akt signaling pathway was enriched in KEGG terms in the RNA-seq and MeDIP-seq data, but whether *Usp7* regulates FGSCs via the PI3K/Akt signaling pathway is unknown and needs further research.

The functional study of FGSCs is of great significance for our understanding of oogenesis and ovarian development. FGSCs were reported to have the capacity to differentiate into oocytes. Many women cannot produce normal eggs because of premature ovarian failure. Applying of FGSCs is a new strategy to solve the problem of female premature ovarian failure. However, there have been few reports about the molecular mechanisms of the proliferation and differentiation of FGSCs. Even less research has been performed about the regulation of FGSCs for an epigenetic perspective. However, epigenetic modification is of great significance in the process of gamete production and embryonic development. In this study, we found that *Usp7* regulates the proliferation and differentiation via DNA methylation.

It has been reported that deubiquitylases (DUBs) act to remove ubiquitin from their protein substrates and protect them from degradation [11]. *Usp7* is a deubiquitylase that is classified into the ubiquitin-specific protease (USP) family. *Usp7* targets various proteins, such as chromatin-associated factors (DNMT1 [8], and UHRF1 [48]), as well as tumor suppressors (p53 [5], PTEN [35]). It is well established that *Usp7* plays diverse roles in genome stability, cell cycle, apoptosis, proliferation, and differentiation. An assay on the knockout of *Usp7* in mice showed that embryonic growth stagnated and death occurred between E6.5 and E7.5 [16]. USP7 associates with KDF1 and regulates skin differentiation through the deubiquitination and stabilization of IKKα [21]. Generating mice with conditional knockout in the ovaries would be a meaningful approach to further define the functions of *Usp7* in mouse ovarian development.

DNA methylation, in which a methyl group is added to carbon 5 of the cytosine in the CpG context, is an essential epigenetic modification. It has been reported to play a vital role in many biological processes, such as the regulation of gene expression [12], X-chromosome inactivation [3], genetic imprinting [33], and disease formation [15]. In particular, the level of genomic DNA methylation was found to be changed in the process of self-renewal and differentiation of stem cells. In germ cells, DNA methylation can be inherited through cell division and transmitted from one generation to the next. Therefore, clarification of the DNA methylation of FGSCs is vital to understand oogenesis. Our study revealed that *Usp7* overexpression induces FGSC differentiation. We deduced that *Usp7* may affect gamete production and embryonic development. However, more work in this field of study is needed.

To the best of our knowledge, our study is the first to evaluate the effects of *Usp7* in FGSCs in vitro, which suggests that *Usp7* is essential for the fate determination of FGSCs by DNA methylation. This study provides novel mechanisms that control FGSC fate and enables deep insights for understanding FGSC development.

## Materials and Methods

### Culture of Female Germline Stem Cells

The FGSCs were cultured as described previously [42, 49, 50]. In brief, FGSCs were cultured on mitomycin C-treated (10 μg/ml, Sigma) mitotically inactivated mouse STO (derived from mouse SIM embryonic fibroblasts, strain SIM, 5 × 10^4^ cells/cm^2^, ATCC) cell feeders. The culture medium for FGSCs was Minimum Essential Medium-alpha (MEM-alpha; Invitrogen, Carlsbad, CA, USA), supplemented with 10% fetal bovine serum (FBS), 10 ng/ml mouse leukemia inhibitory factor (mLIF; Santa Cruz Biotechnology, CA, USA), 10 ng/ml mouse basic fibroblast growth factor (mbFGF; BD Biosciences, Franklin Lakes, NJ, USA), 10 ng/ml mouse epidermal growth factor (mEGF; PeproTech, NJ, USA), 40 ng/ml mouse glial cell line-derived neurotrophic factor (mGDNF; R&D Systems, Minneapolis, MN, USA), 1 mM non-essential amino acids (Invitrogen Life Sciences, MA, USA), 2 mM L-glutamine (Amresco, Lardner, PA, USA), 10 mg/ml penicillin (Amresco), 30 mg/ml pyruvate (Amresco), and β-mercaptoethanol (Sigma-Aldrich, St. Louis, MO, USA). The FGSCs were sub-cultured every 4–6 days at a 1:3 ratio.

### Lentiviral Packaging

For *Usp7*-knockdown lentiviral vectors, we purchased lentivirus packaging plasmids and pGMLV-SC5 lentiviral vectors from Genomeditech Biotechnology Co., Ltd. (Shanghai, China). A puromycin selection site was included for selecting cells in the vector plasmid. We designed a sequence, GCCGAATTAACAGAGAGAAT, as a shRNA target site for *Usp7* knockdown. The blank lentiviral vector was used as the *Usp7*-knockdown control.

For *Usp7-*overexpressing lentiviral vectors, we purchased lentivirus packaging plasmids and pRLenti-CMV-MCS-HA-3Flag-P2A-EGFP lentiviral vectors. We designed a primer sequence for *Usp7* overexpression Primer (F): AAGCTTGTGACGTCTCGGT, Primer (R): GGATCCCCAAAGTTCTAGGC. The blank lentiviral vector was used as the *Usp7*-overexpressing control.

Inhibition or overexpression plasmids and lentivirus packaging plasmids were co-transfected into HEK293T cells to generate lentivirus particles. At 12 h after transfection, enhancing buffer was added. At 48 h after transfection, virus particles were collected by centrifugation.

### Lentiviral Infection

The FGSCs were normally cultured to a confluence of 40% on a 48-well plate and incubated with a 1:1 mixture of culture medium and lentivirus concentrated solution (lentivirus titer: 1 × 10^9^ TU/ml), supplemented with 5 μg/ml polybrene. After 12 h of infection, the cells were cultured with normal FGSC medium for 24 h. Then, they were infected with lentiviral particles and screened with puromycin to obtain *Usp7-*overexpressing or -knockdown FGSCs.

### CCK8 Assay

Lentivirus-infected cells were seeded on a 96-well plate, with 5000 cells per well and 200 μl of culture medium. When the cell density reached 70–80%, CCK8 reagent was added to the plate (20 μl/well), followed by incubation for 2 h at 37 °C and 5% CO_2_. The light absorption value was measured at 450 nm using a microplate reader (Bio-Tek Instruments, Thermo Fisher Scientific, Winooski, VT, USA).

### EdU Assay

The cells were normally cultured to a density of 80% and incubated with 50 μM EdU reagent for 2 h. Then, 4% paraformaldehyde(PFA)was used to fix the cells at room temperature for 30 min, followed by neutralization with 2 mg/ml glycine for 5 min. Next, 0.5% Triton X-100 was used to treat the cells. The cells were supplemented with 1 × Apollo staining solution and incubated for 30 min, followed by washing with PBS containing 0.5% Triton X-100 three times. Then, 1× Hoechst33342 was used to dye the cell nuclei. Images were obtained with a Leica fluorescence microscope.

### Quantitative Real-Time Polymerase Chain Reaction

The total RNA of FGSCs was extracted using Trizol reagent (QIAGEN, Hilden, Germany) and its quality was analyzed using Nanodrop Lite (Thermo Fisher Scientific, Winooski, VT, USA). A total of 1000 ng of RNA was reverse-transcribed into cDNA in a volume of 20 μl using a reverse-transcription kit (Takara, Tokyo, Japan). PCR was performed with Taq DNA polymerase. Quantitative real-time polymerase chain reaction (qRT-PCR) analysis was carried out with SYBR Premix E × Taq (Takara, Shanghai, China) using an Applied Biosystems® 7500 Real-Time PCR System. The data were analyzed by the 2^-ΔΔCt^ method. The primers are shown in Table S1.

### Immunofluorescence Staining

Cells were cultured in a 48-well plate to 80% density. Then, they were washed twice with phosphate buffer saline (PBS) and fixed with 4% PFA for 30 min at room temperature. The cells were blocked with 10% goat serum at 37 °C for 30 min. After washing twice with PBS, the cells were incubated with an anti-MVH antibody in PBS (1:100; Abcam, Cambridge, MA, USA) overnight at 4 °C. The cells were incubated with tetramethylrhodamine isothiocyanate (TRITC)-conjugated secondary antibody in PBS at 37 °C for 1 h (goat anti-rabbit IgG; ProteinTech). Then, the cells were incubated at 37 °C for 5 min with 500 ng/ml 4′, 6-diamidino-2-phenylindole (DAPI). Photographs were obtained using a Leica XP8 fluorescence confocal microscope (Leica, Wetzlar, Germany).

### Western Blotting

The cells were cultured in 35-mm dishes to 90% density. They were then digested with 0.05% trypsin, washed twice with PBS, and lysed with 200 μl of RIPA buffer (Yeasen, Shanghai, China) containing a protease inhibitor cocktail. Protein was obtained by centrifugation (4 °C, 12,000×*g*, 10 min). Protein concentration was measured using a bicinchoninic acid (BCA) protein assay kit (Yeasen, Shanghai, China). After separation by 15% SDS-PAGE gel electrophoresis, the proteins were transferred to polyvinylidene fluoride (PVDF) membranes. Each membrane was blocked with 5% skim milk in Tris-buffered saline with Tween 20 (TBST) for 2 h with shaking at 37 °C. Then, the membrane was incubated with the primary antibody (rabbit-anti-*Usp7*, 1:6000, Abcam; mouse-anti-gapdh, 1:8000, Abcam) at 4 °C overnight. The membrane was then washed three times with TBST for 10 min each time and incubated with the corresponding horseradish peroxidase-conjugated secondary antibody for 1 h at room temperature. Tanon 4600SF (Tanon, Shanghai, China) was used to scan protein bands. The grayscale value of the bands was calculated using ImageJ software.

### RNA-Seq

FGSCs were collected and processed with Trizol reagent (Life Technologies, CA, USA) to extract total RNA. Then, 1000 ng of RNA was used to construct sequencing libraries using the VAHTSTM mRNA-seq v2 Library Prep kit for Illumina1 (Vazyme, Co., Ltd., Shanghai, China). In brief, random hexamer primers were used to synthesize first- and second-strand cDNA, and the cDNA fragments were repaired with the End-It DNA End Repair kit. After adaptor ligation, an A nucleotide was added at the 3′ end of the fragments. The cDNA was amplified by PCR. The library quality was assessed using the Bio Analyzer 2100 (Agilent, Santa Clara, CA, USA). The Illumina HiSeq 2500 platform (Illumina, San Diego, CA, USA) was applied for RNA-seq, and FastQC was used to evaluate the quality of RNA Sequencing (RNA-seq) data.

### Dot Blotting

Genomic DNA was extracted using TIANamp Genomic DNA Kit. Diluted DNA (50 ng/μl) was blotted to an activated PVDF membrane. After cross-linking with a UV cross-linker, the membrane was blocked with 5% skim milk and then incubated with an antibody to 5mc (ab10805, 1:500) at 4 °C overnight. After washing three times with TBST for 10 min, the membrane was incubated with the secondary antibody for 1 h at room temperature and then the dots were scanned with Tanon 4600SF (Tanon, Shanghai, China).

### Methylated DNA Immunoprecipitation Sequence

The work of preparing the MeDIP and DNA libraries was carried out as described previously [26]. In brief, we extracted genomic DNA and cleared the redundant RNA with RNase. Then, the genomic DNA was broken into fragments of about 300 bp. The fragmented chromatin was immunoprecipitated with protein A + G magnetic beads bound with 5-methylcytosine antibody. Methylated DNA immunoprecipitation sequence (MeDIP-seq) and input DNA fragments were end-repaired and A-tailed applying the NEBNext End Repair/dA-Tailing Module (E7442, NEB), followed by adaptor ligation with the NEBNext Ultra Ligation Module (E7445, NEB). The DNA libraries were expanded, followed by deep sequencing with an Illumina HiSeq 2000.

### Chromatin Immunoprecipitation Sequence

The ChIP was carried out in accordance with a previously described protocol [39]. Briefly, cells were cross-linked using 1% formaldehyde, lysed using rotation, and broken using an ultrasonic breaker (Diagenode Bioruptor Pico, Belgium). MAGnify Chromatin Immunoprecipitation System (Thermo Fisher Scientific) was used to perform immunoprecipitation (IP) as per the manufacturer’s instructions with the antibody rabbit anti-H3K27ac (Abcam, Ab4729) or rabbit anti-H3K27me3 (Millipore, 07-449). Chromatin samples were sent for high-throughput sequencing. We used Bowtie2 (v2.3.1) to align the chromatin immunoprecipitation sequence (ChIP-seq) DNA reads to the reference genome. Each ChIP library was compared against the DNA input background library in the corresponding cell type condition. Specifically, histone marks H3K27me3 and H3K27ac in *Usp7* FGSCs and *Usp7-*knockdown FGSC control were compared with the DNA input *Usp7-*knockdown FGSCs and *Usp7-*knockdown FGSC control, respectively. FDR < 0.05 was used to determine ChIP-seq peaks relative to the input libraries.

### Gene Ontology and Kyoto Encyclopedia of Gene and Genomic Pathway Analysis

The biochemical processes involving the differentially expressed mRNAs and genes showing different DNA methylation, H3K27me3, and H3K27ac patterns were elucidated by Gene Ontology (GO) analysis. The signal pathways of the mRNAs and genes were elucidated by Kyoto Encyclopedia of Gene and Genomic (KEGG) analysis. The data were uploaded to DAVID (http://david.abcc.ncifcrf.gov/home.jsp) and the enrichment results were obtained. Fisher’s exact test was applied to evaluate the results, and the false discovery rate (FDR) was used to correct the *p* values (p value <0.05; fold change >2).

### Rescue Assay

These inhibitors (C646, GSK343 and decitabine; MedChemExpress, Shanghai, China) were initially dissolved in 10 mM DMSO to a range of concentrations (C646: 1, 2, 4, 6, 8, and 10 μM; GSK343: 1, 2, 4, 6, 8, and 10 μM; decitabine: 1, 2, 4, 6, 8, and 10 μM) with FGSC medium. The FGSCs were cultured to a density of 40% on a 48-well plate and treated with inhibitor for 24 h. Finally, the appropriate concentrations of different inhibitors (C646: 4 μM; GSK343: 4 μM; decitabine: 8 μM) were determined according to the results of CCK8.

### Statistical Analysis

All experiments in the study were replicated at least three times. The data are presented as mean ± SEM. Student’s *t* test was performed with SPSS software. Differences were considered to be statistically significant at *p* < 0.05.

## Supplementary Information

ESM 1 (PDF 2424 kb)
