# Supplementary material for: Ubiquitin-Specific-Processing Protease 7 Regulates Female Germline Stem Cell Self-Renewal Through DNA Methylation
Source: Stem Cell Rev Rep. 2020 Nov 5;17(3):938–51. doi: 10.1007/s12015-020-10076-9 (PMC8166723; doi:10.1007/s12015-020-10076-9)
Supplement: Supplementary file 1 — (PDF 2424 kb) [file 12015_2020_10076_MOESM1_ESM.pdf]

Supplementary Information for

Ubiquitin-specific-processing protease 7 regulates female germline stem cell self-renewal through DNA methylation

Yongqiang Zhao[1#], Xiaoyong Li[1#], Geng Tian[1#],Xinyan Zhao3#,Jiemin Wong[2], Yue Shen[3], Ji Wu[1, 3*]

[1] Renji Hospital, Key Laboratory for the Genetics of Developmental and Neuropsychiatric Disorders (Ministry of Education), Bio-X Institutes, School of Medicine, Shanghai Jiao Tong University, Shanghai, 200240, China

[2] Shanghai Key Laboratory of Regulatory Biology, Fengxian District Central Hospital-ECNU Joint Center of Translational Medicine, Institute of Biomedical Sciences and School of Life Sciences, East China Normal University, Shanghai 200241, China.

[3] Key Laboratory of Fertility Preservation and Maintenance of Ministry of Education, Ningxia Medical University, Yinchuan, 750004, China

[#] These authors contribute equally to this work

*Corresponding author: Ji Wu, jiwu@sjtu.edu.cn

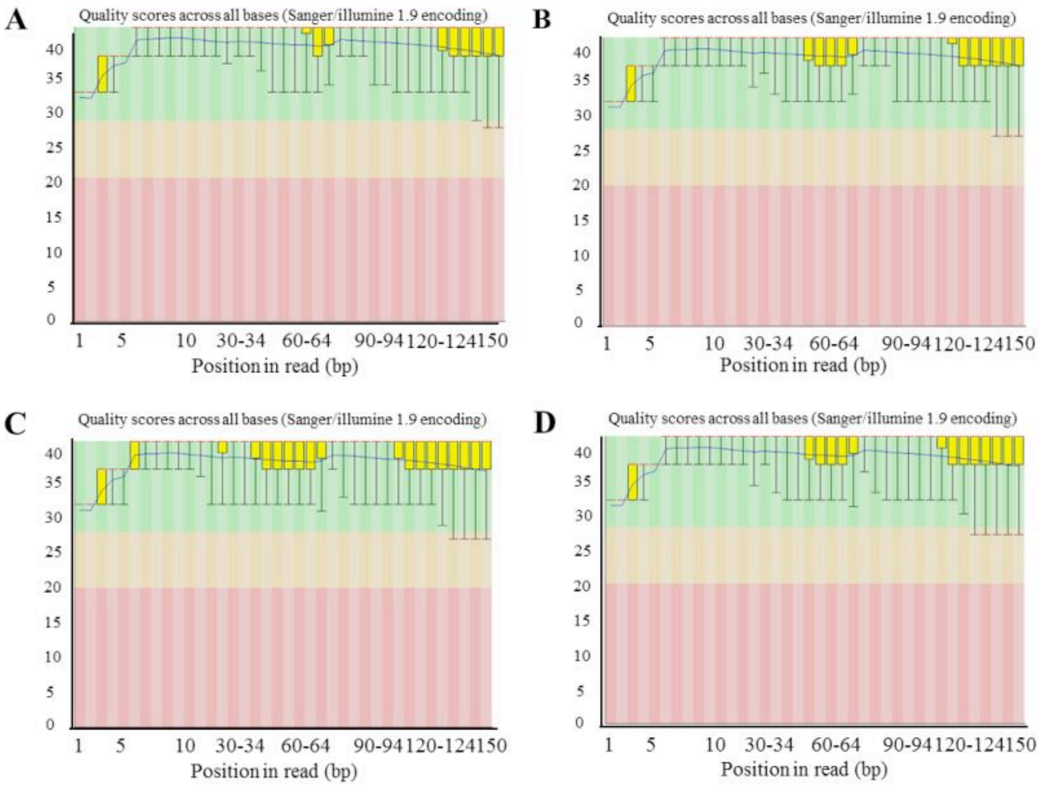

Figure S1. Quality control for RNA-seq data. (A) A representative example of quality control metrics of the RNA-seq reads of *Usp7*-knockdown FGSCs as indicated by FastQC FGSCs. (B) A representative example of quality control metrics of the RNA-seq reads of *usp7*-knockdown FGSC control as indicated by FastQC. (C) A representative example of quality control metrics of RNA-seq reads of *usp7*-overexpressing FGSCs as indicated by FastQC FGSCs. (D) A representative example of quality control metrics of RNA -seq reads of *usp7*-overexpressing FGSC control as indicated by FastQC FGSCs.

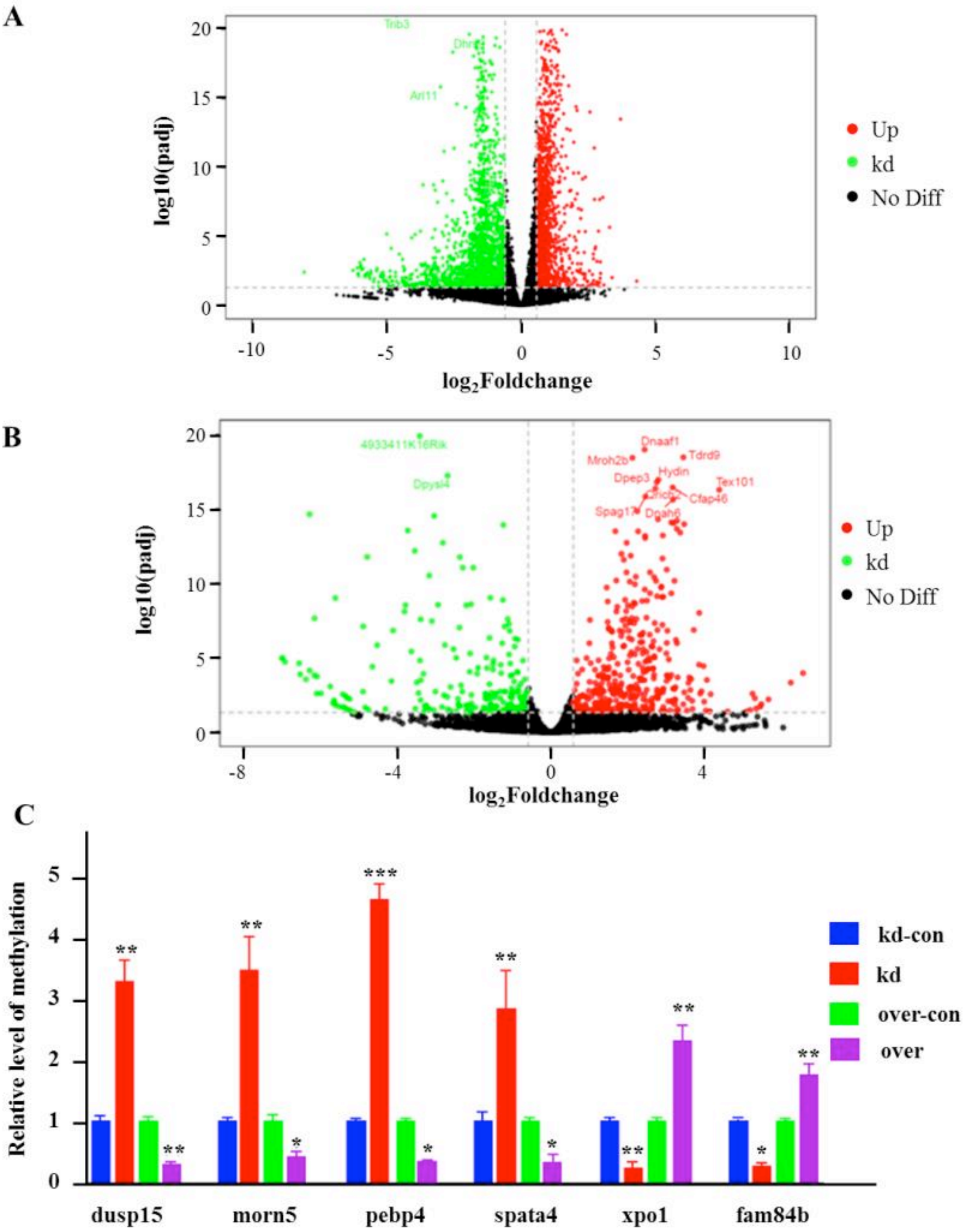

Figure S2. RNA-seq revealed that gene expression was changed by *usp7*. (A) Volcano plots of differentially expressed mRNAs in *Usp7*-knockdown FGSCs and control. (B) Volcano plots of differentially expressed mRNAs in *Usp7*-overexpressing FGSCs and control. (C) RNA-seq data were identified by randomly selected genes: *Dusp15*, *Morn5*, *Pebp4*, *Spata4*, *Xpo1*, and *Fam84b.*

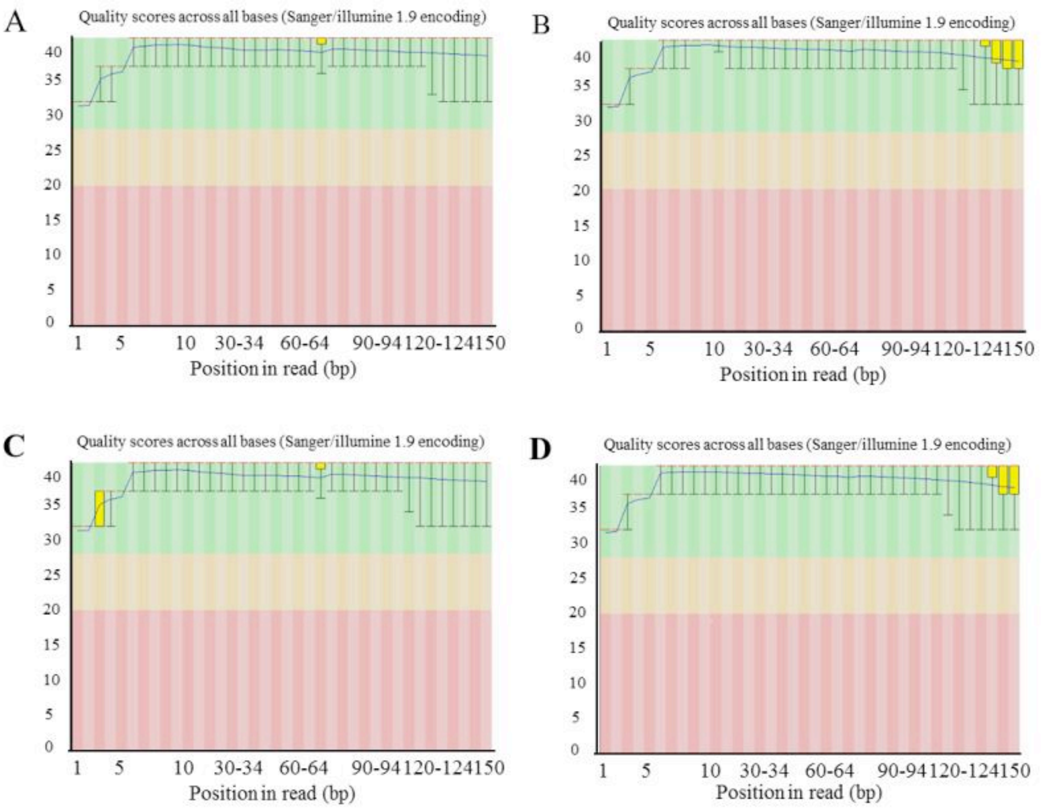

Figure S3. Quality control for MeDIP-seq data. (A) A representative example of quality control metrics of input RNA-seq reads of *Usp7*-knockdown FGSCs as indicated by FastQC FGSCs. (B) A representative example of quality control metrics of immunoprecipitation RNA-seq reads of *Usp7*-knockdown FGSCs as indicated by FastQC FGSCs. (C) A representative example of quality control metrics of input RNA-seq reads of *Usp7*-knockdown FGSC control as indicated by FastQC FGSCs. (D) A representative example of quality control metrics of immunoprecipitation RNA-seq reads of *Usp7*-knockdown control FGSCs as indicated by FastQC FGSCs.

A

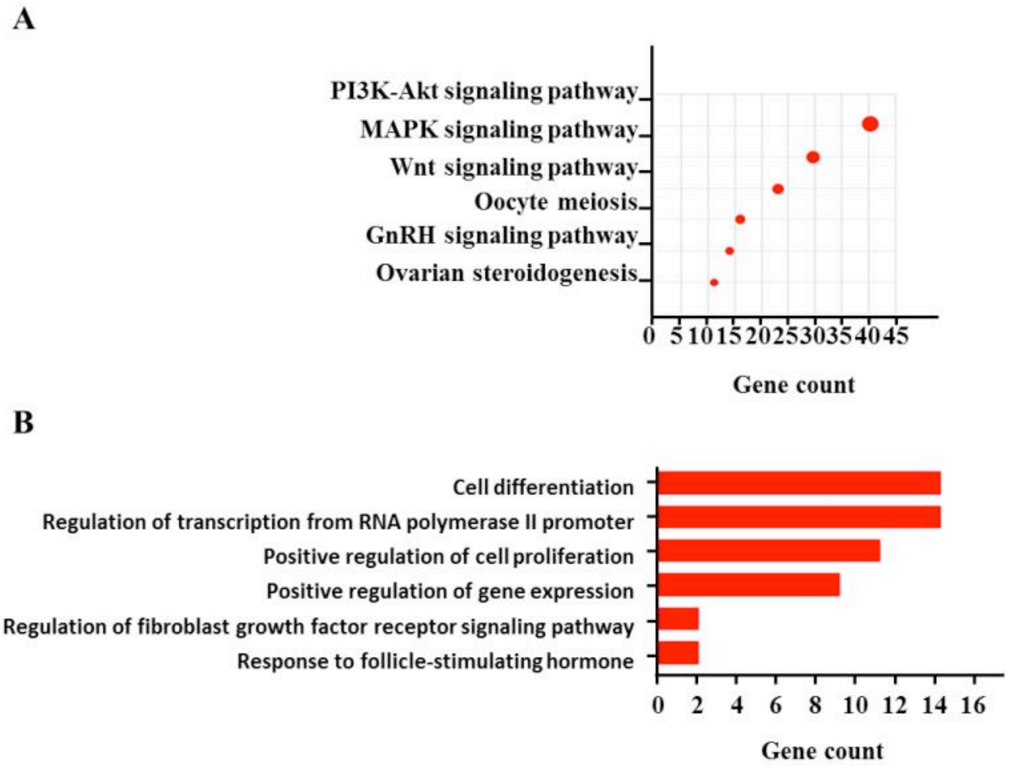

Figure S4. GO and KEGG analyses of MeDIP-seq data. (A) The genes showing different DNA methylation patterns were subjected to KEGG analysis. Terms were selected and shown. (B) The genes overlapping between MeDIP-seq and RNA-seq data were subjected to GO analysis. Terms were selected and shown.

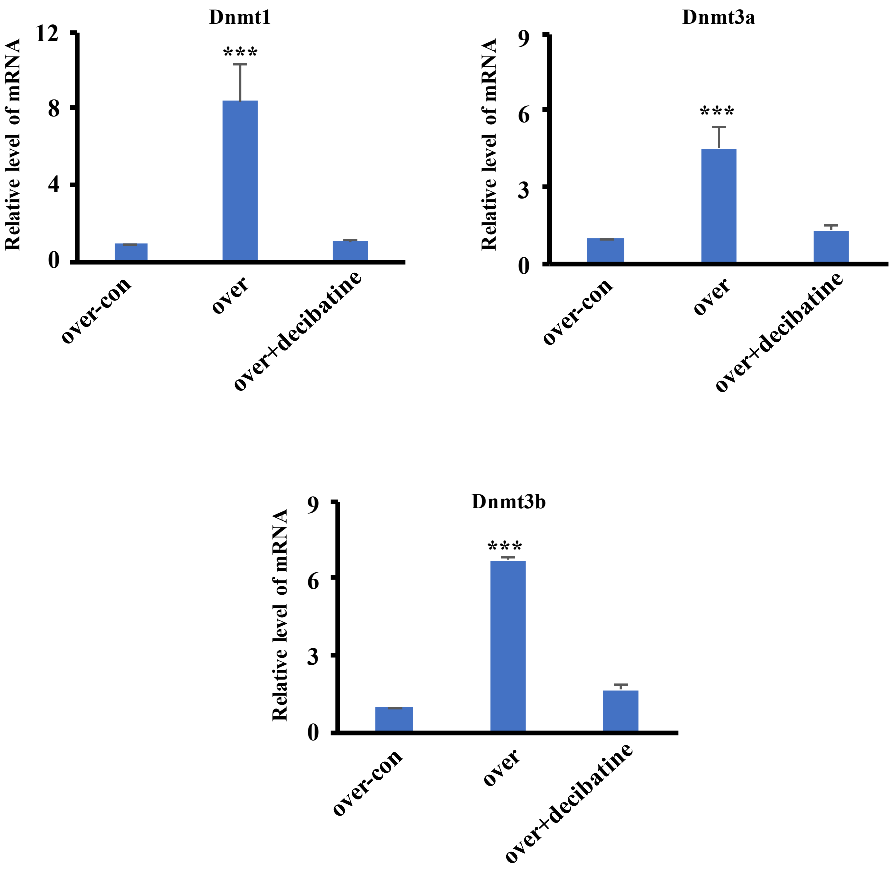

Figure S5. RT-qPCR experiments showed that decitabine worked as expected.

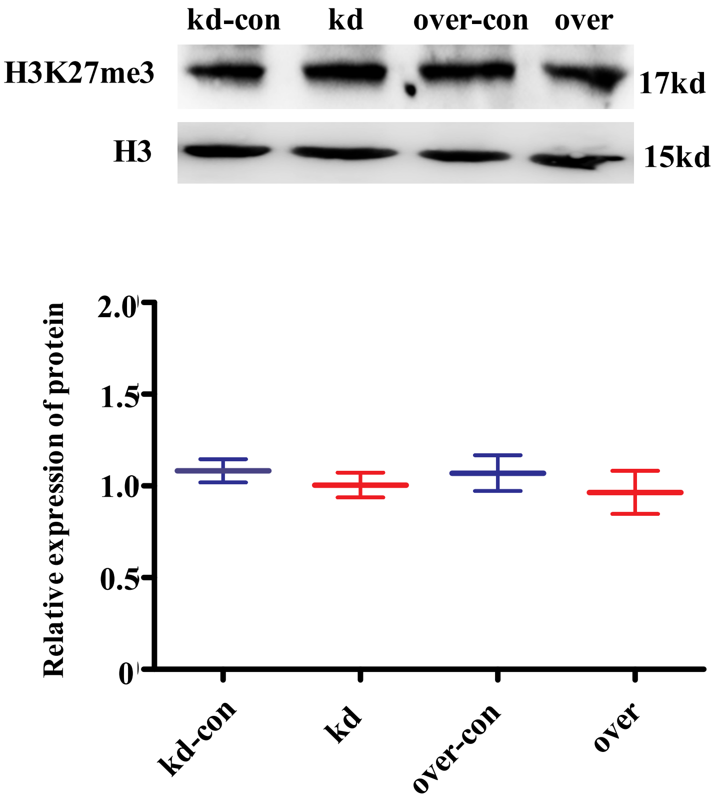

Figure S6. Representative western blots of acid-extracted histones from FGSCs using antibodies recognizing H3K9me3.

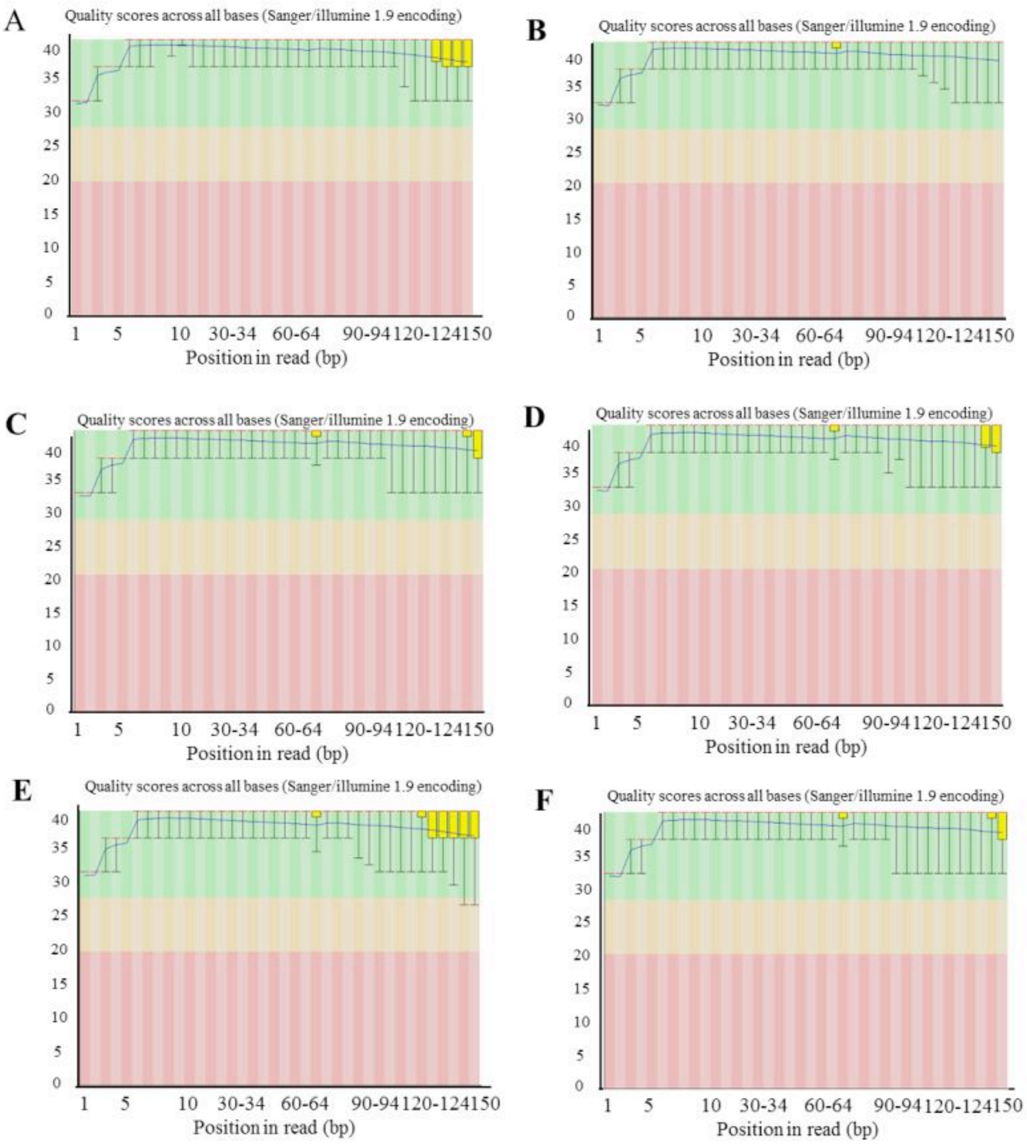

Figure S7. Quality control of ChIP-seq data. (A) A representative example of quality control metrics of input ChIP-seq reads of *Usp7*-knockdown FGSC control as indicated by FastQC FGSCs. (B) A representative example of quality control metrics of input ChIP-seq reads of *Usp7*-knockdown FGSCs as indicated by FastQC FGSCs. (C) A representative example of quality control metrics of H3K27me3 immunoprecipitation ChIP-seq reads of *U*sp7-knockdown FGSCs as indicated by FastQC FGSCs. (D) A representative example of quality control metrics of H3K27me3 immunoprecipitation ChIP-seq reads of Usp7-knockdown FGSC control as indicated by FastQC FGSCs. (E) A representative example of quality control metrics of H3K27ac immunoprecipitation ChIP-seq reads of Usp7-knockdown FGSCs as indicated by FastQC FGSCs. (F) A representative example of quality control metrics of H3K27ac immunoprecipitation ChIP-seq reads of Usp7-knockdown FGSC control as indicated by FastQC FGSCs.

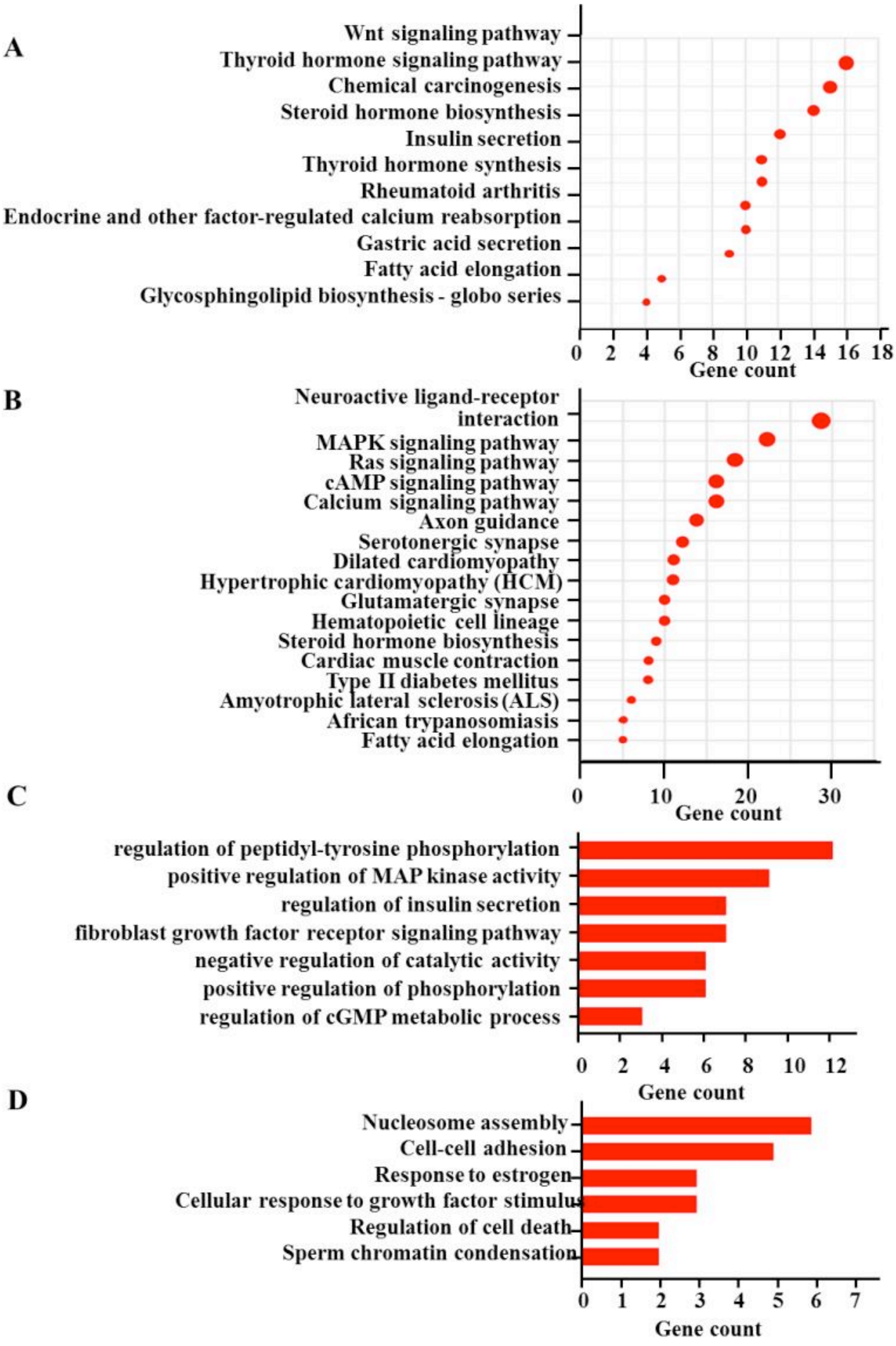

Figure S8. GO and KEGG analyses of ChIP-seq data. (A) The genes showing different H3K27me3 modification patterns were subjected to KEGG analysis. Terms were selected and shown. (B) The genes showing different H3K27ac modification patterns were subjected to KEGG analysis. Terms were selected and shown. (C) The genes overlapping between H3K27me3 ChIP-seq and RNA-seq were subjected to GO analysis. Terms were selected and shown. (D) The genes overlapping between H3K27ac ChIP-seq and RNA-seq were subjected to GO analysis. Terms were selected and shown.

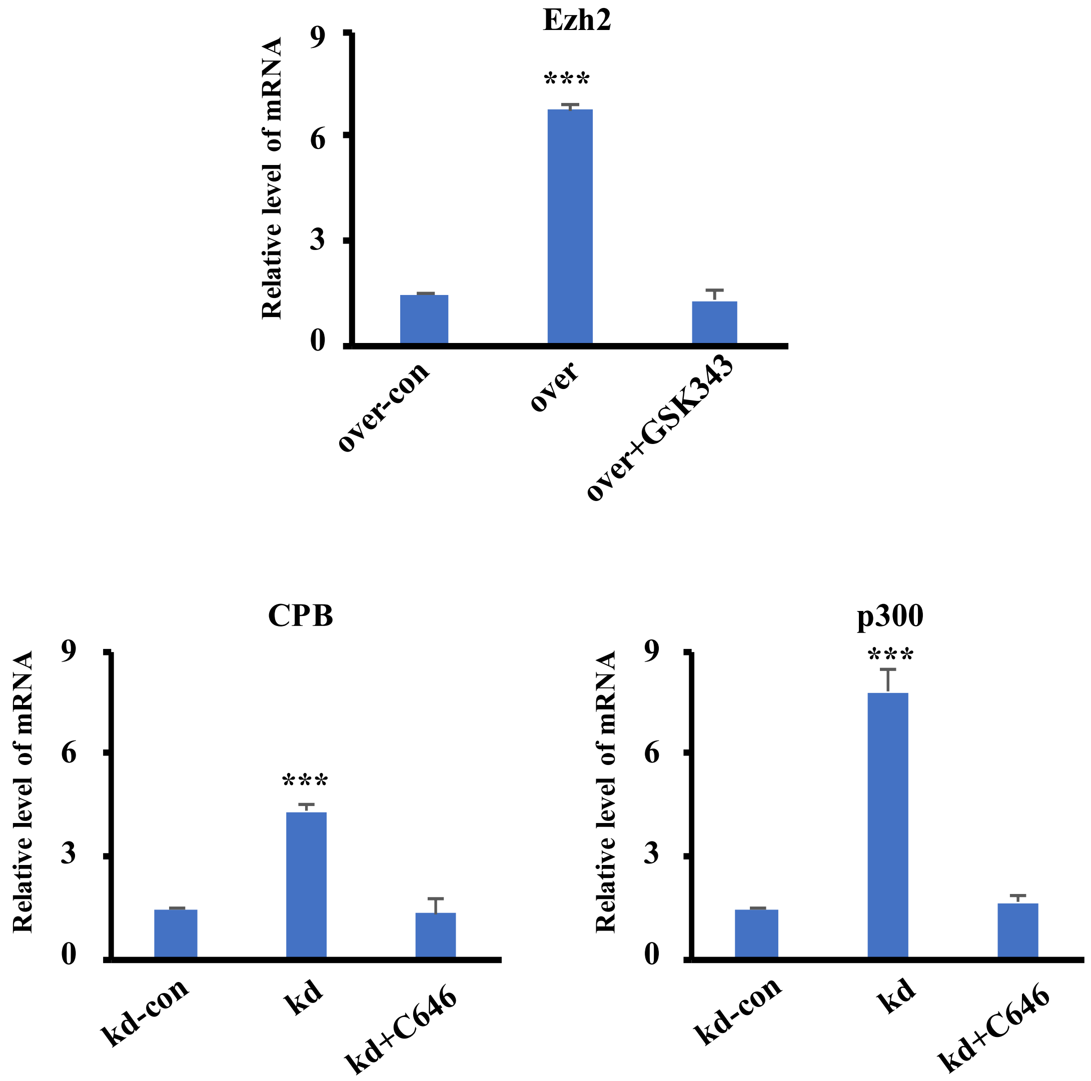

Figure S9. RT-qPCR experiments showed that GSK343 and C646 worked as expected.

Table S1

Primer list for RT-qPCR

stra8	194	F: TTTGACGTGGCAAGTTTCCTG
		R: TAACACAGCCAAGGCTTTTGA
sycp3	185	F: AGCCAGTAACCAGAAAATTGAGC
		R: CCACTGCTGCAACACATTCATA
usp7	227	F: AAGTCTCAAGGTTATAGGGACGG
		R: CCATGCTTGTCTGGGTATAGTGT
pou5f1	132	F: CACCATCTGTCGCTTCGAGG
		R: AGGGTCTCCGATTTGCATATCT
etv5	162	F: TCAGTCTGATAACTTGGTGCTTC
		R: GGCTTCCTATCGTAGGCACAA
foxo1	144	F: GGGTCCCACAGCAACGATG
		R: CACCAGGGAATGCACGTCC
akt	122	F: TGGGTTCAGAAGAGGGGAGAA
		R: AGGGGATAAGGTAAGTCCACATC
gapdh	211	F: TGGATTTGGACGCATTGGTC
		R: TTTGCACTGGTACGTGTTGAT
dusp	114	F: GCATGACCAAGGTACTTCCTG
		R: GGGGTGATTCGTGGATAGAGAT
morn5	123	F: TACACCGGGAGCCAGTATTTC
		R: TTCTCCGTGGAACATGCCG
pebp4	203	F: AGCGGACGAGGATGTTTTCTC
		R: AGCCAACCCAGTTCACTCAC
spata4	145	F: CGCAGCATCAAGACCTACAC
		R: CTGAGTAGCCCTTGTCCTTCA
fam84b	116	F: TGTCTACGTGGGCAATTTCCA
		R: AGCGATACAGATCGTTGACCA
xpo1	160	F: GCCACCACTGGGGTAACTC
		R: CTTCGGGAAAAGTCACAGGG
Primer list for qRT-PCR		
sycp3	163	F: AGACATGGGACATGAAGTAGGC
		R: CCTCTCTCGTTCGTTCGTTTT
gapdh	123	F: AGGTCGGTGTGAACGGATTTG
		R: TGTAGACCATGTAGTTGAGGTCA
blimp	483	F: CGGAAAGCAACCCAAAGCAATAC
		R: CCTCGGAACCATAGGAAACATTC
dazl	524	F: ATACCTCCGGCTTATACAACTGT
		R: GACTTCTTTTGCGGGCCATTT
mvh	283	F: GGCTTCAGACTTCGCCTCC
		R: AACCTGAGGTCCACAGTATGC
oct4	198	F: AGCTGCTGAAGCAGAAGAGG
		R: GGTTCTCATTGTTGTCGGCT
